# Prophylactic cranial irradiation in patients with small-cell lung cancer: the experience at the Institute of Oncology Ljubljana

**DOI:** 10.2478/v10019-010-0038-4

**Published:** 2010-09-09

**Authors:** Karmen Stanic, Viljem Kovac

**Affiliations:** Institute of Oncology, Department of Radiation Oncology, Ljubljana, Slovenia

**Keywords:** small-cell lung cancer, brain metastases, prophylactic cranial irradiation

## Abstract

**Background:**

Prophylactic cranial irradiation (PCI) has been used in patients with small-cell lung cancer (SCLC) to reduce the incidence of brain metastases (BM) and thus increase overall survival. The aim of this retrospective study was to analyze the characteristics of patients with SCLC referred to the Institute of Oncology Ljubljana, their eligibility for PCI, patterns of dissemination, and survival.

**Patients and methods:**

Medical charts of 357 patients with SCLC, referred to the Institute of Oncology Ljubljana between January 2004 and December 2006, were reviewed to determine characteristics of patients chosen for PCI. The following data were collected: age, gender, performance status (PS), extent of the disease, smoking status, type of primary treatment with outcome, haematological and biochemical parameters, PCI use, and finally brain metastases (BM) status at diagnoses and after treatment.

**Results:**

PCI was performed in 24 (6.7%) of all patients. Six (25%) patients developed brain metastases after they were treated with PCI. Brain was the only site of metastases in 4 patients, two progressed to multiple organs. Median overall survival of patients with PCI was 21.9 months, without PCI 12.13 months (p = 0.004). From the collected data there were good prognostic factors: age under 65 years, limited disease (LD), performance status, normal levels of lactate dehydrogenase (LDH) and normal levels of C-reactive protein levels (CRP). Other prognostic factors did not show statistical significant values.

**Conclusions:**

Survival of patients with LD, who have had PCI, was significantly better than those who had not. We decided to perform PCI in patients with LD, in those with complete or near complete response, and those with good performance status (≥ 80). We did not use PCI in extended disease (ED). The reason for that shall be addressed in the future. Doses for PCI were not uniform, therefore more standard approach should be considered.

## Introduction

Small-cell lung cancer (SCLC) expresses aggressive behaviour. Combined treatment with chemotherapy and radiotherapy provides response rates between 50–85% in limited disease (LD). Local recurrence rate decreases with combined treatment; however, brain metastases (BM) become the most common site of relapse. Brain metastases are present in about 20% of patients at the time of diagnosis, but in autopsy findings the rate reached over 50%.^1^,^2^ As in other cancers, in clinical practice BM are diagnosed with computer tomography (CT), less common with magnetic resonance imaging (MRI)^3^,^4^; and all are treated with radiotherapy.^5^

In the early 1970s, prophylactic cranial irradiation (PCI) has been proposed to improve overall survival, because it is well known that central nervous system is relatively refractory to chemotherapy due to the blood-brain barrier. In the 1980s and 1990s there were many prospective studies conducted to investigate the use of PCI; however, only after the publication of two meta-analysis reporting improvement, both, in overall survival and disease free survival, PCI became a part of the standard treatment in SCLC. The first meta-analysis by Auperin *et al.* in 1999 reported the 5.4% increase in the rate of survival at three years as well as the increased rate of disease-free survival.^6^ Meert *et al.* in meta-analysis in 2001 composed 12 randomized trials and reported a hazard ratio of 0.48 for the incidence of brain metastases after PCI.^7^

Recent studies suggest that patients in extensive disease setting could also benefit from PCI.^8^,^9^

The aim of this analysis was to review the use of PCI, to analyze the characteristics of patients with SCLC, referred to the Institute of Oncology Ljubljana, eligibility for PCI, patterns of dissemination, and survival.

## Patients and methods

Cancer Registry of Republic of Slovenia reported 574 newly diagnosed patients with SCLC in the period between 2004 and 2006.^10^–^12^ Three hundred fifty seven patients (62.19%), reviewed in this analysis, were referred for further treatment to the Institute of Oncology Ljubljana, mainly from University Clinic of Respiratory and Allergic Diseases Golnik and University Clinical Centre Maribor. One patient refused all types of further diagnostic procedures and treatments and was excluded from further evaluation.

The following data were collected: gender, age, extent of disease, performance status, smoking status, presence of other malignancies, starting serum levels of haemoglobin (Hb), lactate dehydrogenase (LDH) and C-reactive protein (CRP), type of treatment, response to treatment, PCI information, pattern of dissemination, BM status at diagnoses and after the treatment.

LD included patients with lesions confined to ipsilateral hemitorax, and regional and supra-clavicular lymph nodes. Extended disease (ED) was characterized by an evident and/or proven metastases.

Irradiation was performed at the Institute of Oncology Ljubljana; however, chemotherapy was delivered either at Institute of Oncology Ljubljana (189 patients), University Clinic of Respiratory and Allergic Diseases Golnik (123 patients) or at University Clinical Centre Maribor (29 patients). Twenty one referred patients received no treatment due to poor performance status at presentation at the Institute or due to deterioration of disease during the waiting time for therapy.

Treatment responses were evaluated according to the data available in medical charts as judged by radiation oncologist, based either on X-ray or CT examination during the follow-up. Some of the complete responses (CR) were also confirmed bronchoscopically.

PCI patients were irradiated on Cobalt unit with 1.25 MV or on linear accelerator with 5 or 6 MV photon beams for 5 days per week, once daily. The irradiated field involved whole brain using two opposed lateral fields.

As established the biologic effectiveness of radiation schedules depends on total dose and dose per fraction. The Equivalent Dose in 2-Gy fraction (EQD2) was calculated with the equation as derived from the linear-quadratic model

EQD2=D x [(d+α/β)/2 Gy+α/β)],

where D = total dose, d = dose per fraction, α = linear (first-order dose-dependent) component of cell killing, β = quadratic (second-order dose dependent) component of cell killing, α/β -ratio = the dose where both components are equal. In analysis α/β-ratio of 10 Gy was used to calculate the biological effectiveness of radiation for tumor-cells and α/β -ratio of 3 Gy was used for normal tissue.^13^

### Statistics

Statistical analysis was performed using personal computer and software statistical package SPSS, version 13 (SPSS Inc., USA).

The overall survival time was defined as the time from diagnosis to death or until the end of follow up period on April 1^st^ 2010. The number of surviving patients was confirmed at this date.

Time to progression to brain was defined as time from diagnosis to confirmation of brain metastases by image diagnostics. For patients with PCI time to development of brain metastases was calculated also for period after completion of PCI to confirmation of brain metastases by image diagnostic.

Survival was calculated according to Kaplan-Meier’s method and differences were confirmed by the log-rank test. Independent variables that appeared statistically significant on univariate analysis were tested by multivariate Cox regression analysis model.

## Results

Between January 2004 and December 2006 institutions referred 357 patients with SCLC for further treatment to the Institute of Oncology; 356 were evaluable. Characteristics of patients are detailed in Table 1.

Median age at diagnosis was 61.86 years (40–83); majority were male (76%).

LD was present in 46% of patients, ED in 53%. Performance status, expressed in numbers of the Karnofsky scale, could be collected for majority of patients; however, for 17% patients only descriptions of status could be found in medical records. Majority of patients were smokers (46%). For ex-smokers (24%) qualified patients who stopped smoking at least one year prior to diagnosis. Only 8 (2%) patients were non-smokers; for 28% of patients data could not be retrieved from the medical records. Thirty six (10%) patients have had second malignancy, 6 synchronously and 30 before SCLC. Majority have had head and neck tumours (13), non-SCLC (6), skin tumours including melanoma (6), breast tumours (3), lymphoma (2), prostate carcinoma (2) and other types (3). Two hundred twenty six (63.48 %) patients have had CT or MR imaging during their diagnostic work up procedure – there were 15 (4.2%) without it; for 113 (31.74%) patients, data were not available.

The type of treatment and outcome are presented in Table 2. Majority of patients were treated with chemotherapy and irradiation. Chemotherapy as the only treatment was delivered mainly to patients with ED and 13 patients were irradiated only. Four patients underwent surgery and completed chemotherapy. Treatment resulted in 9 complete responses (CR), 90 partial responses (PR), 71 stable diseases (SD) and 35 progressive diseases (PD). For 150 patients evaluation was not appropriately recorded.

Metastases to brain as the only site of dissemination was present in 37 patients (10.39%) at the time of diagnoses. Twenty-nine patients (8.14%) progressed after primary treatment.

Radiotherapy oncologists proposed PCI to 30 patients, whom they considered eligible, but 6 have refused it. 24 patients (6%) received PCI (20 male and 4 female), mean age of patients with PCI was 53.54 years. Characteristics of patients who received PCI are presented in Table 3. All patients with PCI had LD, statistical significant better performance status, were younger and smokers or ex smokers, only one patient had previous other malignancy.

Dose schedules of PCI were not uniform and are presented in Table 4. No trends in difference of BM frequency with increased biological equivalent dose (calculated as EQD2) received at PCI could be detected.

After PCI 6 (25%) patients developed brain metastases, in 4 patients brain was the only site of metastases, in 2 patients the disease progressed to multiple organs. In 4 out of 6 patients additional cranial irradiation was performed; in 2 patients the disease progressed while waiting for radiotherapy.

Brain was the first site of metastases in 29 patients with LD SCLC; among them 4 patients have had PCI and 25 patients were without PCI, including also 3 patients that have refused PCI. BM were present in 37 patients at the time of diagnosis (ED), 48 patients developed BM later. Overall incidence of BM in our population was therefore 32%.

The mean time to development of BM as a single site of progression for patients with PCI was 32.7 months (14.59–58.62). Mean time to development of BM as single site of progression for 25 other patients with LD who did not have PCI was 10.75 months (0.72–30.1). The difference was statistically significant (p<0.001).

The median overall survival (OS) for all 356 patients with SCLC included in analyses was 9.4 months (95% CI; 8.37 – 10.44)

**FIGURE 1:**
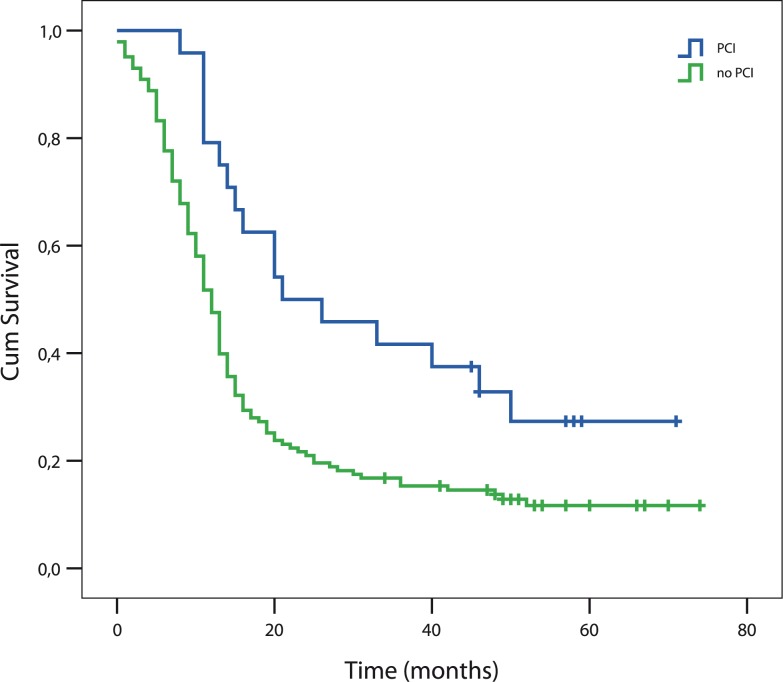
Survival of patients with prophylactic cranial irradiation (PCI) and without PCI (p = 0.004).

The median OS of 167 patients presented with LD SCLC was 13.34 months (95% CI; 12.17–14.51). Median OS of patients with PCI was 21.9 months (95% CI; 6.31–37.48), for those without PCI was 12.13 months (95%CI; 10.69–14.51). The difference was statistically significant (log rank, p=0.004) (Figure 1). On our cut-off date on April 1^st^ 2010 there were 28 patients still alive, 7 of them have received PCI.

Univariate analysis including all patients with SCLC showed statistically significant better survival in patients with age < 65 years, PS > 80, normal LDH and CRP levels, those with PCI and LD and, surprisingly, smokers. In multivariate analysis only LD (p<0.0001, HR = 0.49, 95 % CI 0.332–0.722) and PS (p = 0.03, HR = 0.63, 95 % CI 0.419–0.973) were identified as independent prognostic factors. Since PCI was only performed in patients with LD, separate analysis was performed for this population. In univariate analysis age < 65 years, PS > 80 and PCI showed statistically significant better survival. Multivariate analysis identified only age (p=0.001) and PS (p=0.008) as independent prognostic variables.

## Discussion

PCI has been used in patients with LD SCLC to reduce the incidence of BM and increase overall survival, however reports suggest it should be used also in patients with ED SCLC. In our institution only patients with LD received PCI (14.37%). Retrospective reports in the literature mention about 8%.^14^

Standard treatment consists of combination of chemotherapy and thoracic irradiation of the site of primary tumour.^15^ Combined treatment was delivered to 129 (77.24%) patients with LD SCLC, also the majority of PCI patients in our review were given this treatment; one patient received only chemotherapy and was referred from another institution and one patient underwent only surgery and chemotherapy prior to PCI.

PCI is eligible in patients who achieve complete or near complete response after treatment of primary tumour. In our review only 69 (41.3%) patients in LD group met this criteria; however, data for 60 patients from the same group of LD were not available – the majority of them completed treatment in other institutions and were evaluated there. In group of patients with PCI 5 CR and 17 PR (near CR) were observed, for 2 patients appropriate data were not available in medical records.

None of our patients with ED SCLC received PCI, although 30 had PR responses, however, there were no CR. There are reports that suggest considering PCI also in patients who respond to first line chemotherapy.^16^

Patients who received PCI were younger than SCLC population studied. Radiation oncologists have chosen for PCI patients with the Karnofsky performance status (PS) of 80 or higher. This is in accordance with performance status patient’s selection in prospective studies.^17^ The majority of patients were heavy smokers as was expected in population of patients with SCLC.^18^ Heavy smokers have comorbidities and therefore usually lower performance status, making them less likely candidates for radical treatment and also for PCI.^19^ Bremnes *et al.* reported gender, extent of disease, PS, Hb levels and LDH to be independent prognostic factors.^20^ In our analyses only age < 65 years and PS were independent factors of survival in multivariate analysis.

Doses of PCI in our review were not uniform. Meta analysis suggested trend towards increased reduction of BM rate with increased dose, however, prospective study exploring high versus low dose in PCI found no reduction in total incidence of BM, but there was increased mortality with higher doses.^6^ Therefore a dose of 25 Gy was suggested to be the standard care in LD SCLC.^16^,^21^ All our patients received biological equivalent doses higher than 25 Gy, but no increased mortality nor difference in frequency of BM according to the biological equivalent dose could be detected. The number of analysed PCI patients was small; therefore no conclusions could have been made.

According to our review 4 patients refused PCI. Details of this refusal were not described in our medical records. We could assume that the fear of possible side effects might have been one of the reasons. Several studies reported neurological impairment or abnormalities potentially related was PCI.^9^,^22^–^26^ Acute toxicity consisted mostly of alopecia, headache, fatigue, nausea and vomiting and was usually manageable on outpatient basis. Long term toxicities such as memory loss, intellectual impairment, demenca, ataxia or seizures could be of great concern.

The incidence of BM as the first site of relapse at 5 years have been reported to be 37% in a group of patients not receiving PCI and 20% in PCI group.^17^ However, patients in the study reported had only CR and included also a proportion of ED SCLC. Recent retrospective report indicated 25% incidence of development of BM after PCI, however, number of patients was again small.^27^ The same proportion of patients developed BM also in our series.

There are still doubts among radiation oncologists about using PCI, although even cost effectiveness and quality of life studies beside studies confirming improvement in BM control, OS and DFS have been published.^28^ There are decision making tools and practice guidelines available, but judgment of radiation oncologist should prevail specially in cases of near CR.^29^–^31^

## Conclusions

Our analysis confirmed increased median survival time and decreased incidence for BM in patients with PCI.

Our policy of treatment was to perform PCI in patients with LD and good performance status, the two variables that independently showed better survival. Adding PCI in these patients setting further increased survival. Possibilities of using PCI also in ED SCLC in our institution should be further explored in the future. Doses for PCI were not uniform therefore more standard approach should be considered.

## Figures and Tables

**TABLE 1:** Characteristics of patients

**Number of patients**	**356**	**%**
**Gender**		
Male	270	75.84
Female	86	24.15
**Age (years)**	61.86 (40–83)	
**Clinical stage**		
Limited disease	167	46.10
Extended disease	188	52.80
No data available	1	0.2
**Performance status (Karnofsky)**		
>80	71	19.9
60–80	196	55.05
<60	29	8.14
No data available	60	16.85
**Lactate dehydrogenase (LDH)**		
Normal (≤ 4.23 μkat/L)	158	44.38
Elevated (> 4.23 μkat/L)	102	28.65
No data available	96	26.96
**C-reactive protein levels (CRP)**		
Normal (≤ 15 mg/L)	122	34.26
Elevated (> 15 mg/L)	132	37.07
No data available	102	28.65
**Haemoglobin (Hb)**		
< 120 (g/L)	86	24.15
≥ 120 (g/L)	186	52.24
No data available	84	23.59
**Smoking status**		
Non smokers	8	2.24
Smokers	163	45.78
Ex smokers	84	23.59
No data available	101	28.37
**Other malignancies**	36	10.11
synchronic	6	1.6
metachronic	30	8.4
**Brain metastases (BM) as the only site**	66	18.53
BM at diagnoses	37	10.39
BM after primary treatment	29	8.14

**TABLE 2:** Treatment characteristics and outcome

	**CR**	**PR**	**SD**	**PD**	**unknown**	**All**
**Chemotherapy**	1	22	33	22	67	145
**Chemotherapy and radiotherapy**	6	64	37	13	53	173
**Radiotherapy**	0	3	1	0	9	13
**Surgery and chemotherapy**	2	1	0	0	1	4
**No therapy**						21

CR = complete response, PR = partial response; SD = stable disease; PD = progressive disease

**TABLE 3:** Characteristics of patients with prophylactic cranial irradiation

**Number of patients**		**%**

	**24**	**6.7**
**Gender**		
Male	20	83.33
Female	4	16.66
**Age (years)**	53.54 (43–73)	
**Performance status (Karnofsky)**		
≥ 90	6	25
80	16	66.66
Data not available	2	8.3
**Smoking status**		
Non smokers	0	0
Smokers	16	66.66
Ex smokers	4	16.66
No data available	4	16.66
**Other malignancies**	1	4.1
synchronic	0	0
metachronic	1	4.1
**Lactate dehydrogenase (LDH)**		
Normal (< 4.23 μkat/L)	14	58.33
Elevated (> 4.24 μkat/L)	2	8.3
Data not available	8	33.33
**C-reactive protein levels (CRP)**		
Normal (< 15 mg/L)	10	41.66
Elevated (> 15 mg/L)	8	33.33
Data not available	6	25
**Haemoglobin (Hb)**		
< 120 (g/L)	14	58.33
> 120 (g/L)	3	12.5
Data not available	6	25
**Response to primary treatment**		
CR	5	20.83
PR	17	70.83
Data not available	2	8.33
**Brain metastases**	6	25
As only site of progress	4	16.66
In multiple organ progress	2	8.3

CR = complete response, PR = partial response

**TABLE 4:** Irradiation doses that were applied as prophylactic cranial irradiation

	**Dose schedule**	**EDQ2 α/β=10**	**EDQ2 α/β=3**	**Number of patients treated**	**Number of patients alive**
1	14 x 2.0 Gy = 28.0 Gy	28.0	28.0	3	0
2	15 x 2.0 Gy = 30.0 Gy	30.0	30.0	2	2
3	17 x 2.0 Gy = 35.0 Gy	35.0	35.0	1	0
4	12 x 2.2 Gy = 26.4 Gy	26.84	27.45	3	1
5	13 x 2.2 Gy = 28.6 Gy	29.07	29.74	1	1
6	14 x 2.2 Gy = 30.8 Gy	31.31	32.03	1	1
7	12 x 2.5 Gy = 30.0 Gy	31.25	33.0	4	2
8	14 x 2.5 Gy = 35.0 Gy	36.45	38.5	5	1
9	10 x 3.0 Gy = 30.0 Gy	32.5	36.0	4	0

EDQ2 = Equivalent Dose in 2-Gy fraction
